# Discovery and Biosynthesis of the Novel Glycotetrapeptide Antibiotic Biffamycin A

**DOI:** 10.1002/anie.202511349

**Published:** 2026-05-06

**Authors:** Michael W. Brigham, Edward S. Hems, Daniel C. L. Van, Justin E. Clarke, Sergey Nepogodiev, Julius S. P. Adamson, Christian Bassi, Michael E. Webb, Glyn R. Hemsworth, Orde Q. Munro, Barrie Wilkinson, Ryan F. Seipke

**Affiliations:** ^1^ Astbury Centre for Structural Molecular Biology, School of Molecular and Cellular Biology University of Leeds Leeds UK; ^2^ Department of Molecular Microbiology, John Innes Centre Norwich Research Park Norwich UK; ^3^ Centre For Microbial Interactions Norwich Research Park Norwich UK; ^4^ NMR Platform John Innes Centre Norwich UK; ^5^ Astbury Centre for Structural Molecular Biology School of Chemistry University of Leeds Leeds UK; ^6^ School of Chemistry University of Leeds Leeds UK

**Keywords:** amino acid hydroxylase, glycopeptide antibiotics, natural product biosynthesis, silent gene clusters, *Streptomyces*

## Abstract

The clinical deployment of antibiotics is undermined by antimicrobial resistance. Without new agents to treat antibiotic‐resistant bacterial infections, mortality rates are predicted to reach 10 million people per year by 2050. Most antibiotics are derived from natural products (NPs) produced by bacteria; however, this resource was abandoned by industry because of high rediscovery rates. We are amid a natural product renaissance fuelled by inexpensive access to genome sequencing and sophisticated bioinformatic tools, which have highlighted that most of the biosynthetic pathways for NPs are not expressed in the laboratory. Here, we engineered the expression of a silent biosynthetic gene cluster harboured by an environmental isolate of *Streptomyces albidoflavus*. Using a bioinformatics‐guided approach, we isolated and structurally characterised a novel glycopeptide antibiotic (GPA) named biffamycin A, which is the smallest GPA known and harbours unprecedented 5‐chloro‐4‐methoxy tryptophan and 3‐hydroxy(α‐D‐mannoysl)‐D‐lysine moieties. Biffamycin A possesses antimycobacterial and antistaphylococcal bioactivity, including against methicillin‐ and vancomycin‐resistant *Staphylococcus aureus*.

## Graphical abstract

Genetic de‐regulation of a silent biosynthetic pathway allowed isolation and characterisation of a novel glycopeptide antibiotic named biffamycin A, which harbours unprecedented 5‐chloro‐4‐methoxy tryptophan and 3*R*‐hydroxy(α‐D‐mannoysl)‐D‐lysine moieties and is bioactive against MRSA and VRSA.

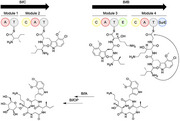

## 1 Introduction

A key challenge in antimicrobial drug development is the discovery of agents that circumvent clinically prevalent resistance mechanisms, which are particularly problematic for the ESKAPE group of pathogens (*Enterococcus faecium, Staphylococcus aureus, Klebsiella pneumoniae, Acinetobacter baumannii, Pseudomonas aeruginosa* and *Enterobacter* species). Most antibiotics in use are derived from microbial natural products, particularly those produced by *Streptomyces* bacteria and other filamentous Actinobacteria [1]. *Streptomyces* species harbour many biosynthetic pathways, but only a handful of them are typically productive in a laboratory setting [2]. Accessing the chemical diversity encoded by such biosynthetic pathways is widely believed to be the best route to a second golden era of antibiotic discovery.

Nonribosomal peptides (NRPs) are a structurally complex and diverse family of natural products that often exhibit therapeutically relevant activities. NRPs are produced by multifunctional enzymes called nonribosomal peptide synthetases (NRPSs), which are large assembly line‐like machines organised into modules whose biochemical function is to incorporate a single monomeric building block into the growing polypeptide. NRPS biosynthetic modules can be grouped into two categories: loading modules and elongation modules [3]. A loading module generally consists of two active domains, an adenylation (A) domain that activates an amino acid substrate and loads it onto the second domain, a peptidyl carrier protein (PCP) domain, which possesses a 4′‐phosphopantetheinyl prosthetic group to which the growing peptide chain is covalently linked. Elongation modules also harbour A and PCP domains but additionally contain a condensation (C) domain, which precedes the A domain (C‐A‐PCP) and catalyses peptide bond formation between two PCP‐bound peptide units. Both loading and elongation modules can harbour additional tailoring domains, such as epimerase (E) domains, amongst others, that modify peptide intermediates. The terminal elongation module usually possesses a C‐terminal thioesterase (TE) domain, which transfers the polypeptide intermediate from the final PCP domain onto a conserved Ser residue, after which either a hydrolytic or macrocyclisation reaction occurs to produce the mature peptide or depsipeptide.

Genome mining enabled us and others to discover a novel standalone peptide cyclase (SurE) from the surugamide biosynthetic system [4, 5, 6]. SurE is the archetypal member of what has been dubbed the penicillin‐binding protein (PBP)‐TE family, orthologues of which are widespread in *Streptomyces* species, with about 15% of sequenced strains harbouring at least one NRPS system with a SurE offloading strategy [4]. Our continued characterisation of the surugamide‐producing strain *S. albidoflavus* S4 [4] led us to identify a silent/cryptic NRPS system that, instead of harbouring a standalone SurE cyclase, contains a *cis‐*encoded (i.e., embedded) SurE domain at the C‐terminus of the terminal biosynthetic module, a unique observation within NRPS megaenzymes. This intriguing observation was the impetus for a targeted campaign to access the product of this pathway, which is revealed here to be a novel glycotetrapeptide antibiotic with unprecedented 5‐chloro‐4‐methoxy tryptophan and 3‐hydroxy(α‐D‐mannoysl)‐D‐lysine moieties.

## 2 Results and Discussion

### 2.1 Identification and Analysis of the Biffamycin (*bif*) Biosynthetic Gene Cluster (BGC)

**FIGURE 1:**
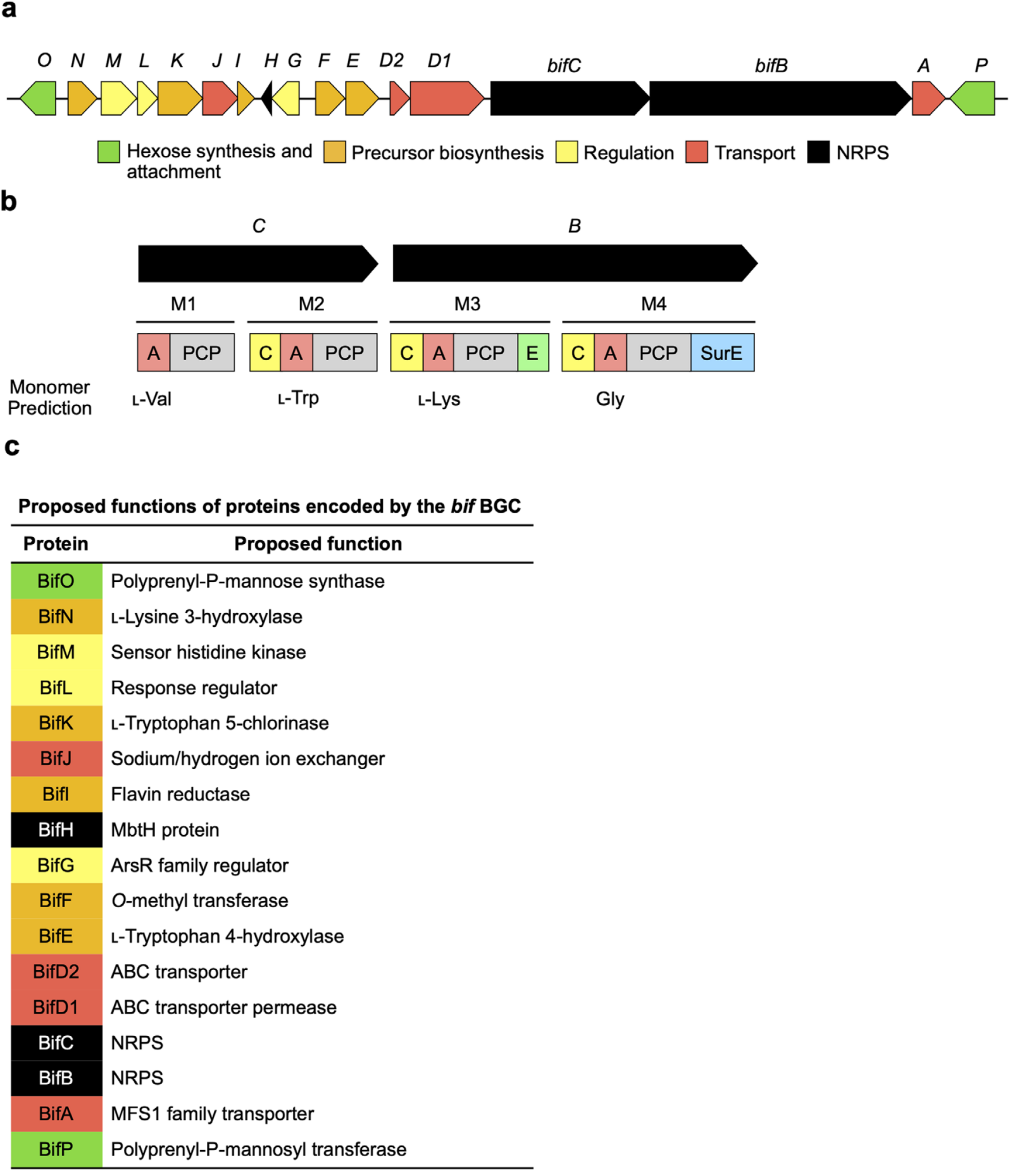
The biffamycin *(bif)* biosynthetic gene cluster (BGC). (a) Gene organisation of the *bif* BGC, colour‐coded by deduced function as indicated. (b) Biosynthetic module organisation of BifC and BifB. A, adenylation domain; T, thiolation domain; C, condensation domain; E, epimerase domain; SurE, macrocyclisation domain. Domain organisation was predicted using antiSMASH v6.1.1 [14], and A‐domain specificities were predicted using PARAS [15]. (c) Proposed function of proteins encoded by the *bif* BGC based upon bioinformatic analyses and experiments performed during this study. The *bif* BGC is available under GenBank accession PV425438.

The *bif* BGC harboured by *Streptomyces albidoflavus* S4 [7] is composed of 17 genes (*bifABCD1D2EFGHIJKLMNOP*) predicted to encode the production of a modified glycotetrapeptide (Figure 1, Table S1). Bioinformatic analyses were used to predict the putative product of the *bif* BGC, which served as a framework for its experimental identification. This framework considered the following: the two dimodular nonribosomal peptide synthetases, BifC (domain organisation: A1‐PCP1‐C1‐A2‐PCP2) and BifB (domain organisation: C2‐A3‐PCP3‐E3‐C3‐A4‐PCP4‐SurE) assemble a peptide scaffold, which, based on analysis of the predicted adenylation domain active site residues using PARAS (Figure S1) [8], is predicted to be cyclo[ʟ‐Val‐ʟ‐Trp‐D‐Lys‐Gly]. The terminal biosynthetic intermediate is likely cyclised by a domain that shows high predicted structural similarity to a standalone peptide cyclase named SurE (Figures S2 and S3) [4, 5, 6]. The presence of an E‐domain within the third module (Figure S4) indicates an amino acid in the D‐configuration is likely present at the third position of the final molecule. The resulting cyclotetrapeptide scaffold is predicted to be mono‐hexosylated by a putative hexosyl transferase (BifP), the substrate for which is provided by the polyprenyl‐P‐hexose synthase (BifO). Analysis of other proteins encoded within the BGC indicated that two amino acids, one basic polar, and the other aromatic, are likely modified either on‐board or prior to activation by their cognate A‐domains. BifEF share 49% and 60% sequence identity with OhmKJ, respectively, which were recently proposed to introduce a methoxy group to the Trp moiety of ohmyungsamycin [9], while BifKI constitutes a predicted Trp halogenase/flavin reductase pair. BifN is a putative α‐ketoglutarate (α‐KG)‐dependent amino acid hydroxylase with 41% shared amino acid identity to VioC, a structurally characterised standalone ʟ‐Arg 3*S*‐hydroxylase [8, 10, 11]. Thus, BifEFIK likely generates Cl‐MeO‐Trp, and BifN may hydroxylate Lys, the presumed substrates for the second and third biosynthetic modules, respectively. Taken together, these analyses allowed us to predict that biffamycin would consist of a glycopeptide composed of hexosylated cyclo[ʟ‐Val‐ʟ‐(Cl‐MeO‐Trp)‐(3‐OH‐D‐Lys)‐Gly].

### 2.2 Deregulation of the *bif* BGC and Chemical Analysis of Biffamycin A

**FIGURE 2:**
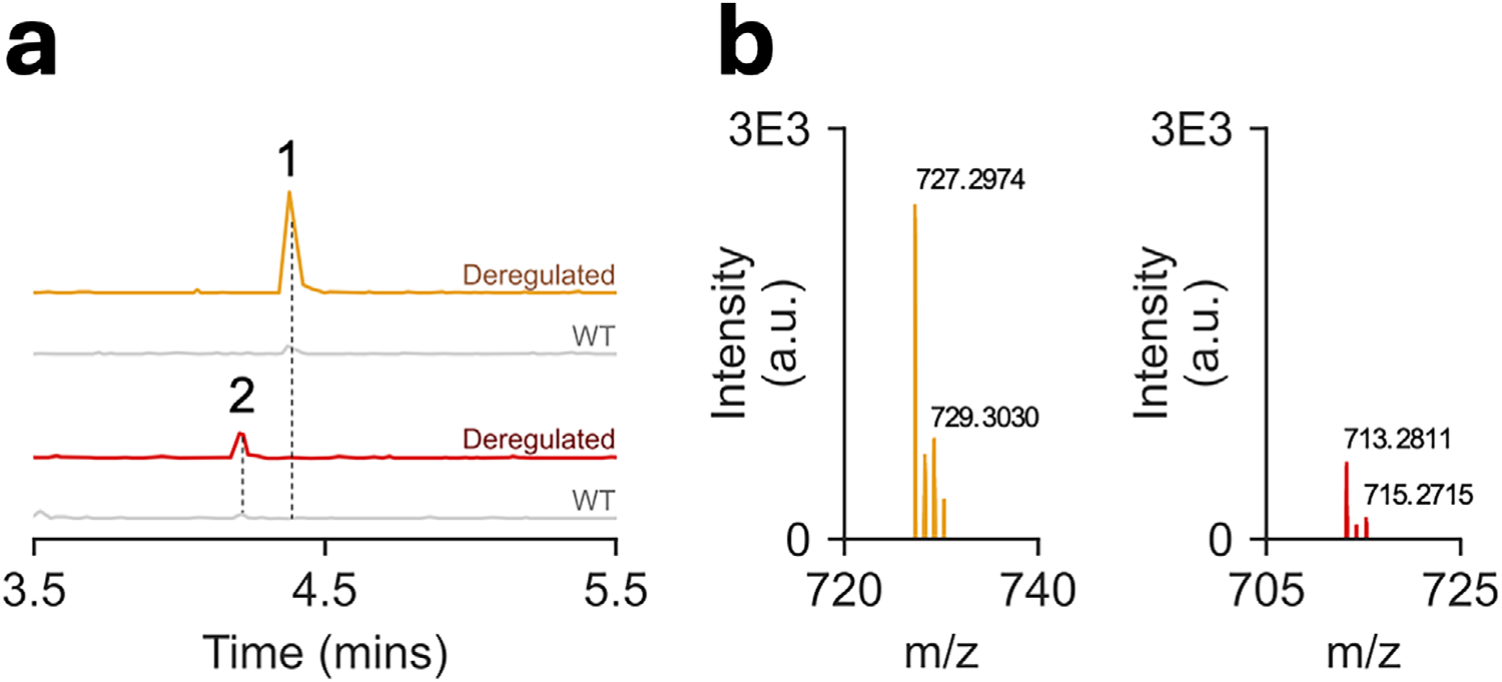
De‐regulation of the *bif* BGC results in production of biffamycins. (a) Production of biffamycin A (**1**) and B (**2**) in minimal medium (MM) by either the WT or the deregulated *S. albidoflavus* S4∆5 ∆*bifO‐bifD1*/pBiff strain. Displayed are extracted ion chromatograms (EICs) for the monoisotopic masses corresponding to the [M + H]^+^ adduct for **1** (C_32_H_47_ClN_6_O_11_) ± 0.01 and **2** (C_31_H_45_ClN_6_O_11_) ± 0.01. (b) Mass spectra for **1** (left) and **2** (right); a.u., arbitrary units. Colours coordinated with companion Figure S6.

To access the product(s) of the *bif* BGC, we cultivated a variant of *S. albidoflavus* S4 (*S. albidoflavus* S4∆5, in which five endogenous BGCs are inactivated [12]), on a small suite of agar growth media (see Methods). Methanolic extracts were prepared from these cultures, concentrated, and analysed by LC‐HRMS. Minute quantities of two metabolites broadly consistent with the molecular weight associated with our bioinformatic prediction were detected in the extract prepared from minimal medium (MM)‐grown culture; however, low production titre precluded their detailed analysis (Figure 2a). Thus, a promoter engineering strategy was used to de‐regulate transcription of the *bif* BGC. The genes *bifO‐*to‐*bifD1* were replaced with a hygromycin resistance gene harbouring an ermE* promoter to facilitate constitutive expression of *bifCBA* on the chromosome of *S. albidoflavus* S4 ∆5 (∆*bifO‐bifD1*). Next, an integrative plasmid harbouring the genes *bifPOKJIFENHD2D1* constitutively expressed from ermE* and *rpsL(XC)* promoters was constructed and introduced into the ∆*bifO‐bifD1* strain to create ∆*bifO‐bifD1*/pBiff (Figure S5). The ∆*bifO‐bifD1*/pBiff strain was cultivated in MM as above, and crude methanolic extracts were prepared and analysed by LC‐HRMS. De‐regulation of the *bif* BGC led to reliable detection of two compounds with an isotopic distribution pattern consistent with a singly chlorinated species, and a mass consistent with the predicted hexosylated compound ((calc. *m/z* 713.2908, obs. *m/z* 713.2811, [M + H]^+^ C_31_H_45_ClN_6_O_11_) and (calc. *m/z* 727.3064, obs. *m/z* 727.2974, [M + H]^+^ C_32_H_47_ClN_6_O_11_)) (Figure 2a,b). The latter compound was the more abundant product and therefore was named biffamycin A (**1**) and is the focus of this study; we named the former compound biffamycin B (**2**). It is common for NRPS systems to produce a small suite of congeners differing in their aliphatic amino acid content [13], so we hypothesised the mass increase of *m/z* 14.0066 for **1** compared to our bioinformatic prediction indicated that the compound may harbour an amino acid with an additional methylene group compared to Val, presumably Ile or Leu. To simplify purification, we sought to determine if we could skew the production profile of **1** relative to **2** by supplementing growth media with specific amino acids. ∆*bifO‐bifD1*/pBiff was therefore cultivated in MM supplemented with either Val, Ile or Leu. Interestingly, supplementation of MM with Ile or Leu led to a marked increase in **1** and no detectable **2,** and supplementation of MM with Val led to increased **2** and only a trace level of **1** (Figure S6). Taken together, these data establish that the *bif* BGC encodes production of at least two metabolites whose production can be modulated by the composition of culture media. As we could not disambiguate whether **1** possessed Ile or Leu by mass spectrometry alone, we chose to characterise **1** from cultures supplemented with Ile.

### 2.3 Purification and Structure Elucidation of Biffamycin A (**1**)

To purify **1**, the producer organism was first cultivated in 8 L of MM supplemented with 5 mM Ile production medium. Following centrifugation and filtration, the clarified supernatant was passed through a C_18_ flash chromatography cartridge to capture **1,** after which the retained components were eluted. The resulting fractions were analysed by LCMS, and those containing target compounds were further purified by preparative HPLC to yield biffamycin A (**1**, 3.5 mg), *des‐*hydroxy‐biffamycin A aglycon (**3**, 180 µg), the precursor 5‐Cl‐4‐MeO‐ʟ‐Trp (**4**, 15.6 mg) and an acetylated derivative, *N*‐acetyl‐5‐Cl‐4‐MeO‐ʟ‐Trp (**5**, 4.4 mg). In addition, the cell pellet was extracted with methanol, and the resulting extract was purified by preparative HPLC to yield the biffamycin A aglycon (**6**, 230 µg) and an additional **3** (230 µg).

**FIGURE 3:**
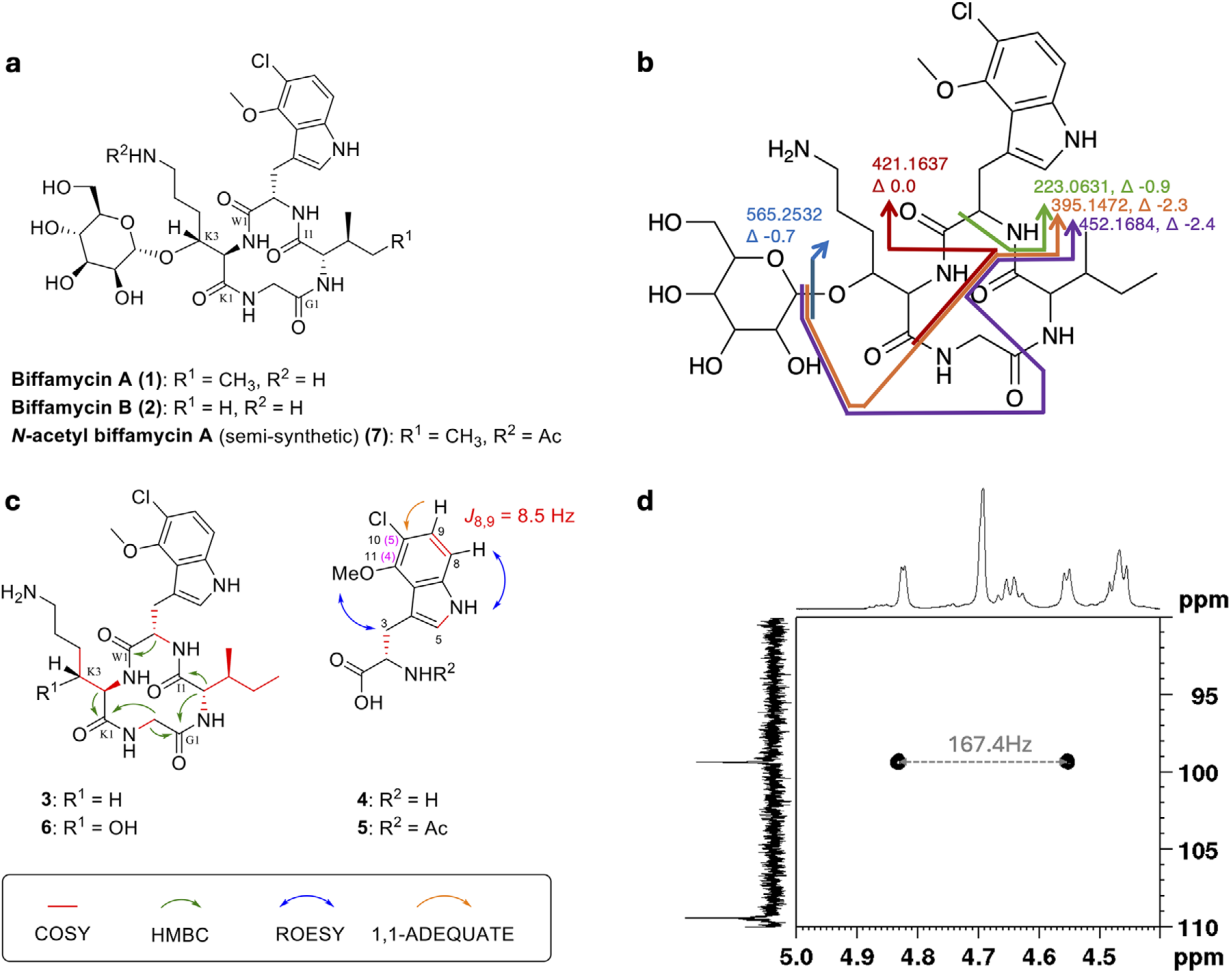
Chemical characterisation of **1**. (a) Structures of biffamycin A (**1**), biffamycin B (**2**) and semi‐synthetic *N*‐acetyl‐biffamycin A (**7**). (b) The LCHRMS^2^ fragmentation pattern for **1** showing the *m/z* and error (ppm) of key fragments used to determine amino acid connectivity. (c) Key 2D NMR correlations used in the structural assignment of compounds **3**–**6**. The IUPAC numbering for the indole ring in **4** and **5** is shown in magenta. (d) Zoomed coupled HSQCed spectrum of **7** showing the anomeric signal as a doublet with ^1^ *J*_CH_ = 167.4 Hz.

Unfortunately, **1** did not have sufficient solubility in common deuterated solvents for NMR analysis, and, as such, the structure of **1** was elucidated using complementary approaches. First, we considered the LCHRMS^2^ data for **1**, **3,** and **6** (Figure 3a; Figures S7 and S9, S10; Tables S2 and S4, S5). **1** gave reliable in‐source fragmentation across several MS instruments with a loss of *m/z* 162, characteristic of a hexose moiety.

Comparison of the MS^2^ spectra of **1** with that of **6** showed that after the loss of a hexose, the fragmentation data were essentially identical. This gave strong evidence that **1** and **6** share the same tetrapeptide backbone, with **1** being glycosylated with a hexose. The predicted connectivity of the cyclotetrapeptide backbone of **1** from bioinformatics was supported by detailed MS^2^ analysis of both **1** and **6,** as well as the full assignment of the 1D and 2D NMR spectra of **6** (Figure 3b; Figure S34–S40; Table S7). Here, COSY was utilised to assign the Gly, Ile and 3‐OH‐Lys side chains of **6**. The HMBC cross peaks between the α‐hydrogens of Gly and the amide carbon of 3‐OH‐Lys and the α‐hydrogen of Ile and amide carbon of Gly established the (3‐OH‐D‐Lys)‐Gly‐ʟ‐Ile sequence which, in combination with MS^2^, confirmed the amino acid connectivity as cyclo[ʟ‐Ile‐(5‐Cl‐4‐MeO‐ʟ‐Trp)‐(3‐OH‐D‐Lys)‐Gly]. Using the same MS^2^ analysis methods suggested a structure for **3** of cyclo[ʟ‐Ile‐(5‐Cl‐4‐MeO‐ʟ‐Trp)‐D‐Lys‐Gly] (Figure 3b; Figures S10–S16; Table S6). Comparison of the ^1^H and ^13^C NMR of **6** and **3** showed great similarities between the corresponding NMR spectra except for a clear downfield shift at the C3 resonance of the D‐Lys residue for **6** (*δ*_H_ 3.75, *δ*_C_ 68.9) when compared with **3** (*δ*_H_ 1.63 & 1.50, *δ*_C_ 29.4), indicating **6** is hydroxylated at the C3 on the D‐Lys side chain. The stereochemistry of this hydroxyl group could not be resolved unambiguously from the NMR data alone, and in all cases the stereochemistry of D‐Lys was inferred from the bioinformatic analysis.

Next, the regiochemistry of the Cl and MeO substituents on the presumed NRPS substrate **4** was assigned by NMR (Figure 3b; Figure S20–S26; Table S7). First the aromatic protons were considered, with a singlet at *δ*_H_ 7.21 assigned to H5 and two doublets at *δ*_H_ 7.15 and *δ*_H_ 7.07 with a coupling constant of 8.5 Hz suggesting a pair of protons with an *ortho* orientation on the indole ring. Next, analysis of the ROESY NMR showed an interaction between the indole N*H* proton at *δ*_H_ 11.21 and the aromatic doublet of H8 at *δ*_H_ 7.15, which indicated the *ortho* aromatic protons were on C8 and C9 (Figure S26). Additional ROESY cross‐coupling between the methoxy protons (H13) and H2 and H3’ of the tryptophan group indicated the methoxy group was on C11 (*δ*_C_ 149.3). Further analysis by 1,1‐ADEQUATE NMR showed a cross coupling between H9 and C10 (*δ*_C_ 115.8 ppm), indicating the position of the chlorine atom (Figure S25). Taken together, **4** was assigned as 5‐chloro‐4‐methoxy‐ʟ‐tryotophan using IUPAC nomenclature. *N*‐acetyl‐5‐chloro‐4‐methoxy‐ʟ‐tryptophan (**5**) was characterised in an analogous manner (Figure 3b; Figures S27–S33; Table S7).

To determine the hexose moiety on biffamycin A (**1**), 50 µg of **1** was hydrolysed with 1 M aqueous trifluoracetic acid. The liberated hexose was identified as mannose by co‐injection/migration of standards by high‐performance anion‐exchange chromatography with pulsed amperometric detection (HPAEC‐PAD) to be mannose (Figure S12). We are unaware of a microbial source of ʟ‐mannose [16] and therefore presume mannose to be in the D‐configuration.

Finally, to address the problem of solubility in NMR solvents, a sample of **1** was *N*‐acetylated using acetic anhydride in pyridine to give **7**, which was partially soluble in DMSO‐d_6_ (Figure 3a; Figures S41–S47; Table S7). LCHRMS^2^ analysis of **7** confirmed the compound was *N*‐acetylated on the 3‐hydroxy‐D‐lysine side chain (Figure S11 and Table S6). The solubility of **7** allowed us to analyse the mannose glycoside. HMBC was used to determine that **7** was glycosylated at the hydroxyl group on C3 of the D‐Lys side chain, with a cross‐peak between the mannose anomeric proton (*δ*_H_ 4.70) and K3 (*δ*_C_ 75.2) (Figure S45). To determine the anomeric configuration of mannose, we ran a coupled HSQCedited spectrum to measure the one‐bond carbon‐proton coupling constants. For the anomeric carbon, ^1^ *J*_CH_ = 167.4 Hz, which would suggest an α‐D‐mannopyranose residue on **7** (Figure 3) [17]. For comparison, we also ran the coupled HSQCedited spectrum for methyl α‐D‐mannopyranoside in DMSO‐d_6_ for which the anomeric carbon gave ^1^ *J*_CH_ = 167.5 Hz (Figure S48). Thus, the final proposed structure of **1** is assigned as: cyclo[ʟ‐Ile‐(5‐Cl‐4‐MeO‐ʟ‐Trp)‐(3‐O‐α‐D‐mannosyl‐D‐Lys)‐Gly].

### 2.4 Conformational Analysis and Stereochemical Assignment

**FIGURE 4:**
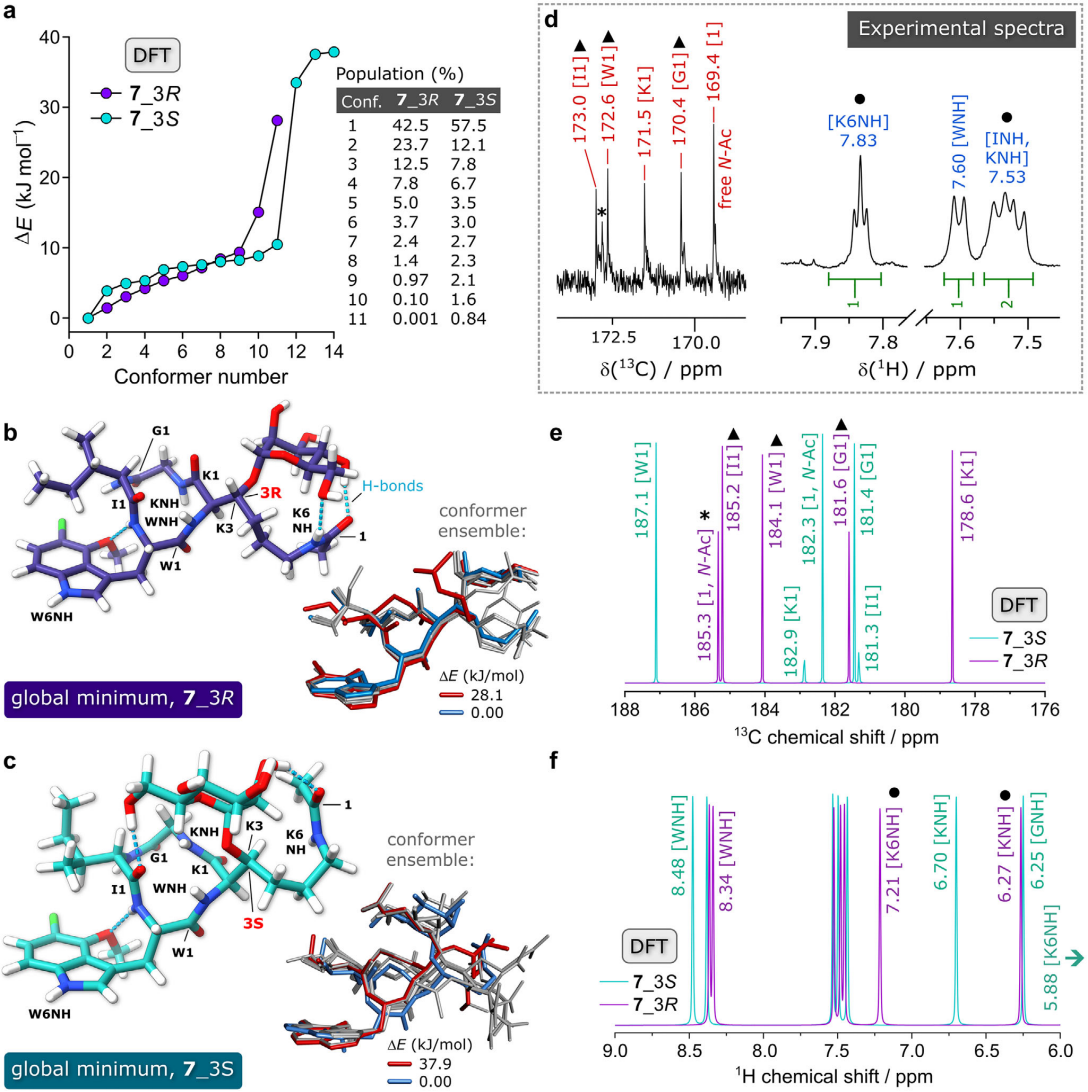
Conformational landscape and NMR properties for **7**. (a) Boltzmann populations and energy distribution for the unique conformers of **7**_3*R* and **7**_3*S* calculated at the r^2^SCAN‐3c level of theory (298.15 K, DMSO solvent continuum). The global minimum energy conformers for the two diastereomers are shown in Parts (b) and (c) along with the NMR atom labelling scheme and superpositions of the structures belonging to each ensemble. Diastereomer **7**_3*R* is more rigid than **7**_3*S*. Despite this, the highest energy conformer of **7**_3*R* exhibits torsional inversion about the lysine amide group (K1) leading to the same macrocyclic ring conformation populated by all conformers of **7**_3*S*. Diagnostic regions in the DFT‐calculated carbon and proton NMR spectra for the two diastereomers are shown in Parts (e) and (f) for comparison with the experimental NMR spectra in Part (d). The black symbols track key matching peaks between theory and experiment.

To resolve the assignment of the stereochemistry of 3‐OH‐D‐Lys (carbon K3) from the NMR data, we performed a stochastic search of the complete conformer space for [**1**_3*R*]^+^, [**1**_3*S*]^+^, [**6**_3*R*]^+^, [**6**_3*S*]^+^, **7**_3*R*, and **7**_3*S* in an implicit water solvent continuum (for **1**) and DMSO (for **6** and **7**) using density functional theory (DFT), specifically the GOAT/GFN2‐xTB [18, 19] conformer search algorithm implemented recently in ORCA 6.1.0 [20, 21]. These searches located between 1175 and 2800 conformers within 25 kJ mol^−1^ of the lowest‐energy conformer for the structures of interest. From the Boltzmann populations of each ensemble, conformers with populations above 1% at the GFN2‐xTB level of theory were selected for geometry optimisation with r^2^SCAN‐3c [22] (triple‐zeta quality basis set) to give final ensembles of accurate structures suitable for high‐level calculation of the NMR shielding tensors (GIAO method, B3LYP/6‐311+G**) [23, 24]. Next, after pruning all equivalent conformers (defined as those with energy differences < 0.20 kJ mol^−1^, Figure 3a–c) we calculated the population‐weighted average ^1^H and ^13^C NMR chemical shifts for comparison with the experimental data (Figure 4d–f). The presence of the mannose ring and *N*‐Ac group of **7** introduced sufficient complexity to the conformer landscapes of **7**_3*R* and **7**_3*S* to permit discrimination of the stereoisomers from the calculated and observed proton and carbon chemical shifts.

As shown in Figure 4, the experimental ^13^C NMR spectrum best matches the DFT‐calculated population‐weighted average ^13^C NMR spectrum of **7**_3*R* in the low field region around 170–180 ppm where the signals of the cyclic peptide carbonyl carbon atoms (not the *N*‐Ac group, which has several rotamers and whose magnetic environment will be substantially affected by H‐bonding with the solvent) exhibit a distinct spectral pattern. Specifically, the experimental chemical shift order I1 > W1 > G1 is equivalent to that calculated for **7**_3*R*, but *notably different* to that for **7**_3*S* (W1 > G1 ≈ I1). Interestingly, the DFT‐calculated resonance for K1 at 182.9 ppm for **7**_3*S*, which has an inverted K1 amide group relative to **7**_3*R*, is seen to occur in precisely the correct chemical shift region between W1 and G1 of **7**_3*R* to give the “ideal” chemical shift pattern, I1> W1> K1> G1, that is an exact match for the experimental signal pattern. From the conformer ensemble of **7**_3*R* (Figure 4b), the highest‐energy conformer (11) has an inverted K1 amide group akin to the global minimum structure of **7**_3*S* and thus a K1 chemical shift that is 2.2 ppm *downfield* (181.1 ppm) of the weighted average chemical shift (178.6 ppm) for this carbonyl carbon. Such a downfield shift upon torsional inversion of the amide C═O group (K1) is significant and in the correct direction (to lower frequency). Because the DFT simulations use an *implicit* solvent continuum model, the experimental conformer distribution could differ from the in silico distribution and comprise a sufficient weighting from conformers with an inverted cyclic tetrapeptide ring structure. This might account for the observed discrepancy between the chemical shift of K1 in the experimental and DFT‐calculated ^13^C NMR spectra for **7**_3*R*. This aside, the key point here is that the DFT calculations for **7**_3*R* give a 75% match to the experimental carbon chemical shift pattern for **7** (3 signals out of 4) without invoking K1 torsional inversion for the rather rigid—and thus diagnostically important—macrocyclic core of the compound.

Analysis of the proton NMR spectra (Figure 4f) also confirms the best match between the DFT‐calculated NMR data for **7**_3*R* and the experimental spectrum (Figure 4d). Specifically, the chemical shift order for K6NH and KNH *exactly matches* the experimental order (K6NH > KNH) with good enough agreement in the absolute chemical shifts (within 0.6 ppm of the experiment for K6NH) to assign the stereochemistry of the diastereomer and confirm that the paired H‐bonding of the *N*‐Ac group to the mannose –CH_2_OH group and its adjacent OH group is significant. (Conformers 1, 2, and 5 make up 71% of the ensemble population and display the dual H‐bond shown in Figure 4b.) Regarding the **7**_3*S* diastereomer of **7**, the mannose ring for the 11 lowest‐energy conformations (populations > 0.8%, Figure 4a) is always positioned above the macrocyclic core of the molecule, giving a ‘closed’ conformation, as seen for the global minimum energy structure (Figure 4c). This conformation is maintained by H‐bonding of the Ile amide carbonyl oxygen (I1) to the mannose –CH_2_OH group. The *N*‐Ac‐lysine residue is thus free to adopt many rotamers because the mannose –CH_2_OH group is unavailable for H‐bonding. Consequently, the DFT‐calculations predict an upfield resonance for the *N*‐Ac group's amide proton (5.88 ppm) since it is not stabilised and deshielded by the rigid H‐bonding seen for **7**_3*R*. The chemical shift order for KNH and K6NH in diastereomer **7**_3*S* is thus the *reverse* of what we observe experimentally, firmly pointing to **7**_3*R* as the stereoisomer present in the experimental spectra.

**FIGURE 5:**
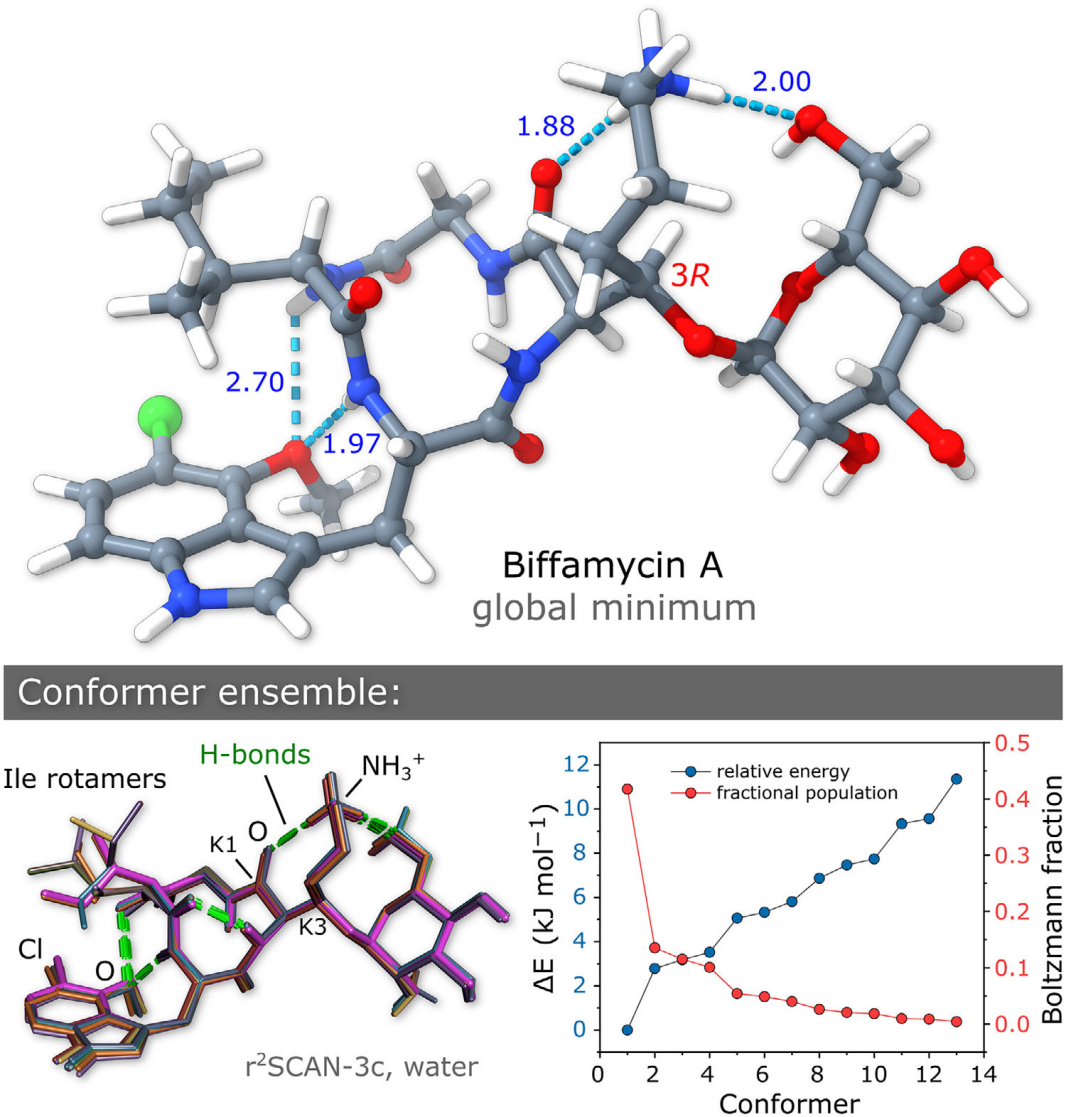
DFT‐calculated structure of **1**_3*R* (biffamycin A) and its conformer distribution at 298.15 K. The global minimum is coloured pink in the ensemble (bottom left) and represents 42% of the conformer population.

Importantly, the DFT calculations highlight the marked conformational differences and substantial changes to the conformer populations for **7**_3*R* and **7**_3*S* caused by the stereochemistry at carbon 3 in the lysine residue (K3) for **7**. Our combined assessment of the experimental and theoretical data for **7** (the semi‐synthetic analogue of **1**) allows us to assign the stereochemistry for biffamycin A as **1**_3*R* with a confidence level approaching 90% (75% from the ^13^C NMR data and 100% from the proton NMR data). This assignment is consistent with a key, yet rare, example of a natural product harbouring 3‐OH‐Lys known as HV‐toxin M, a host‐specific toxin‐related compound produced by the phytopathogenic fungus *Helminthosporium victoriae* [25]. In this cyclic hexapeptide, the stereochemistry for C‐3 of 3‐OH‐lysine is also uniquely 3*R* [26]. The global minimum energy conformation of biffamycin A, specifically in its protonated form [**1**_3*R*]^+^, calculated at the r^2^SCAN‐3c level of theory in water (CPCM model) [27] is shown in Figure 5.

The protonated lysine amino group of **1**_3*R* plays a pivotal role in restricting conformational freedom of the cyclic tetrapeptide by forming a strong bridge between the K1 carbonyl oxygen and the mannose –CH_2_OH group. The significant conformations of **1**_3*R* located in the conformational search, refinement, and pruning workflow (populations > 0.4%) differ structurally from the global minimum by less than 2%. Most of the deviation in the ensemble is associated with three main isoleucine rotamers. In comparison with **7**, biffamycin A is the most rigid and highly stabilised congener in the series with 5 intramolecular hydrogen bonds, including a significant dual H‐bond to the methoxy group's O atom. This “locked‐in” tertiary structure for **1**_3*R* identified by the DFT simulations is clearly enabled by the unusual modifications seen for the indole ring of tryptophan (OMe) and the third carbon of lysine (K3, O–mannose), which might well be critical for its bactericidal activity. However, as discussed later, the in vivo target of this remarkable compound remains to be determined.

### 2.5 Reconstitution of 5‐Cl‐4‐MeO‐ʟ‐Trp and 3R‐OH‐ʟ‐Lys Biosynthesis

**FIGURE 6:**
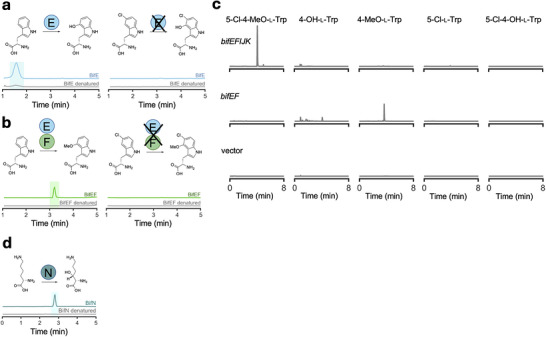
Reconstitution of 5‐Cl‐4‐MeO‐ʟ‐Trp and 3*R*‐OH‐ʟ‐Lys biosynthesis. (a) In vitro reconstitution of 4‐OH‐ʟ‐Trp biosynthesis by BifE. Shown is the LCMS analysis of reaction mixtures of BifE or heat‐inactivated BifE incubated with ʟ‐Trp (left) or 5‐Cl‐ʟ‐Trp (right); shown are EICs for the monoisotopic mass corresponding to the [M + H]^+^ adduct derived from 4‐OH‐ʟ‐Trp (*m/z* 221.1 ± 0.5) or 5‐Cl‐OH‐ʟ‐Trp (*m/z* 255.1 ± 0.5), respectively. (b) In vitro reconstitution of 4‐MeO‐ʟ‐Trp biosynthesis in a one‐pot reaction of BifEF or heat‐inactivated BifEF with Trp (left) or 5‐Cl‐ʟ‐Trp (right); shown are EICs for the monoisotopic mass corresponding to the [M + H]^+^ adduct derived from 4‐MeO‐ʟ‐Trp (*m/z* 235.1 ± 0.5) or 5‐Cl‐4‐MeO‐ʟ‐Trp (*m/z* 269.1 ± 0.5), respectively. (c) *S. coelicolor* M1152 heterologous expression of 5‐Cl‐4‐OH‐ʟ‐Trp biosynthetic genes as indicated; other pairwise combinations (*bifEIJK, bifFIJK, bifIJK*) are shown in Figure S52. Shown are EICs for the monoisotopic mass corresponding to the [M + H]^+^ adduct for the compound indicated. 5‐Cl‐4‐MeO‐ʟ‐Trp (*m/*z 269.0687 ± 0.01); 4‐OH‐ʟ‐Trp (*m/z* 255.0531 ± 0.01); 4‐MeO‐ʟ‐Trp (*m/z* 235.1077 ± 0.01); 5‐Cl‐ʟ‐Trp (*m/z* 239.0582 ± 0.01); 5‐Cl‐4‐OH‐ʟ‐Trp (*m/z* 254.0458 ± 0.01). MS^2^ fragmentation mapping of 5‐Cl‐4‐MeO‐ʟ‐Trp and 4‐MeO‐ʟ‐Trp produced by M1152 is shown in Figure S55. (d) In vitro reconstitution of 3*R*‐OH‐ʟ‐Lys biosynthesis by BifN or heat‐inactivated BifN with ʟ‐Lys; the EIC corresponds to the [M + H]^+^ adduct for Fmoc‐3*R*‐OH‐ʟ‐Lys (*m/z* 385.2 ± 0.5). For (b) and (d), the *X* denotes that the indicated enzyme(s) do not accept 5‐Cl‐ʟ‐Trp as a substrate.

The chemical structure of **1** revealed the presence of two modified amino acid residues (5‐Cl‐4‐MeO‐ʟ‐Trp, and 3*R*‐OH‐D‐Lys) in the cyclotetrapeptide scaffold. We purified the former from culture supernatant and elucidated its structure above. To the best of our knowledge, chloro‐methoxy‐tryptophans have not previously been reported from a natural or synthetic source; we therefore sought to reconstitute the biosynthesis of 5‐Cl‐4‐MeO‐ʟ‐Trp (**4**) in vitro. However, despite considerable effort, the BifK halogenase could not be produced as a soluble protein from *E. coli*. Thus, we established the biosynthetic origin of **4** through a combination of in vitro reconstitution and in vivo heterologous expression studies. (His)_6_‐BifE was heterologously produced and purified from *E. coli* (Figures S49 and S50), and the hydroxylation activity of BifE and heat‐inactivated BifE was assessed in vitro using ʟ‐Trp and commercially available 5‐Cl‐ʟ‐Trp, which established BifE utilises Trp and not 5‐Cl‐ʟ‐Trp as a substrate (Figure 6a). Next, (His)_6_‐BifF was overproduced and purified from *E. coli* (Figures S49 and S51), and a one‐pot reaction was developed to successfully reconstitute the methoxylation of ʟ‐Trp in vitro (Figure 6b). This enzymatic route for ʟ‐Trp methoxylation is intriguing; while tryptophan hydroxylases are known to catalyse oxygenation of the indole ring in animals, an equivalent enzyme in bacteria remained enigmatic until recently. During the biosynthesis of **1**, C4 oxygenation of ʟ‐Trp is catalysed by a rare heme‐dependent Trp hydroxylase (BifE) that belongs to a new sub‐family of the Trp‐2,3‐dioxygenases reported while our study was in progress [28].

Through sequence analysis, BifK appears to be a conventional flavin‐dependent Trp halogenase, which is well studied [29, 30, 31]. To assess the halogenation activity of BifK, varying combinations of *bifEFJIK* were introduced into *Streptomyces coelicolor* M1152, which lacks the *bif* BGC. Metabolites were extracted from the resulting M1152 heterologous expression strains and analysed for the presence of **4** and relevant pathway intermediates by LCHRMS. The results are displayed in Figure 6c and Figure S52 and indicate that expression of *bifEF* is sufficient for the heterologous production of 4‐MeO‐ʟ‐Trp and that expression of *bifEFIJK* is essential for the heterologous production of **4**. The putative sodium/hydrogen exchanger encoded by *bifJ* was not essential for production of 4‐MeO‐ʟ‐Trp, at least not in *S. coelicolor* M1152. 4‐OH‐ʟ‐Trp was not observed, even in the absence of *bifE*, suggesting 4‐OH‐ʟ‐Trp is either unstable during cultivation or possibly consumed by another physiological process. Interestingly, 5‐Cl‐ʟ‐Trp was not observed in any experiment, suggesting that the BifK halogenase exhibits a tight specificity for 4‐MeO‐ʟ‐Trp. Taken together, these data support a model in which BifE hydroxylates C4 of ʟ‐Trp, the product of which is then methylated by BifF prior to halogenation at C5 by BifK and its cognate reductase partner BifI.

Hydroxylation of Lys C3 is presumed to be carried out by BifN, which is an ⍺‐KG‐dependent amino acid hydroxylase, an enzyme type that is well‐known within NRPS pathways [32, 33] and for which there are two subfamilies: those that work on‐board with the NRPS assembly line (i.e., act on PCP*‐S‐*bound intermediates) and those that act on free amino acids [11, 34].

Our bioinformatics analysis of BifN suggested these two enzyme classes can be resolved phylogenetically (Figure S53), which led us to hypothesise that BifN acts upon free ʟ‐Lys. To experimentally test this hypothesis, (His)_6_‐BifN was overproduced and purified from *E. coli* (Figures S49 and S54) and incubated with ʟ‐Lys alongside a heat‐inactivated control. Reactions were derivatised with fluorenylmethyoxycarbonyl (Fmoc) to enable more reliable chromatography prior to analysis by LCMS. As anticipated, Fmoc‐OH‐ʟ‐Lys was detected for reaction mixtures containing (His)_6_‐BifN but not heat‐inactivated (His)_6_‐BifN (Figure 6d). Taken together, these data suggest that BifN acts on free ʟ‐Lys and does not act on an NRPS‐bound substrate during biosynthesis. For **1**, the BifN‐catalysed 3*R*‐hydroxylation of ʟ‐Lys is critical because it is the attachment point for the D‐mannose. It is interesting to note that only a trace amount of (**3**) was observed during fermentation of the producer strain, which suggests that either BifB^A3^ has a substrate preference for 3*R*‐OH‐ʟ‐Lys and/or BifB^C4^ may have a gatekeeping function to minimise incorporation of ʟ‐Lys. It also suggests that the **3** may not be a substrate for the aglycone exporter.

### 2.6 BifB^SurE^ and BifOP Are Essential for the Production of **1**

Intriguingly, the biffamycin A scaffold is predicted to be cyclised by an embedded SurE cyclase domain harboured within the terminal biosynthetic module of BifB. SurE is a promiscuous peptide cyclase belonging to the newly identified family of PBP‐TEs [4, 5, 6], which have thus far only been observed as standalone enzymes [4, 35]. To experimentally determine if the BifB^SurE^ domain was essential for biffamycin A production, we deleted the BifB^SurE^ coding sequence in the ∆*bifO*‐∆*bifD1* strain using CRISPR‐Cas9 editing and reintroduced the pBiff plasmid (∆*bifO‐bifD1*∆*bifB^surE^*/pBiff). The resulting mutant strain and its parent were cultivated under biffamycin A production conditions, and chemical extracts were analysed by LC‐HRMS. As anticipated, biffamycin A was only detected in the extract generated from the parental strain and absent from the ∆*bifO‐bifD1*∆*bifB^surE^*/pBiff strain, indicating BifB^SurE^ is essential for biffamycin A production (Figure S56).

The D‐mannose moiety is essential for potent antibacterial activity of biffamycin A (Table 1, discussed below). To experimentally determine if the putative polyprenyl‐P‐mannose synthase (BifO) and polyprenyl‐P‐mannosyl transferase are essential for biffamycin A production, we cultured ∆*bifO‐bifD1*/pBiff‐temp‐4 (which lacks *bifOP*) under biffamycin A production conditions. LC‐HRMS analysis of chemical extracts prepared from these cultures revealed the absence of *bifOP* abrogated the production of biffamycin A (Figure S57).

**TABLE 1:** Minimum inhibitory concentration^a^ of **1** and intermediates against microorganisms and human cells.

Organism	Biffamycin A (µg mL^−1^)	Biffamycin A aglycon (µg mL^−1^)	*des‐*hydroxy biffamycin A aglycon (µg mL^−1^)	Vancomycin (µg mL^−1^)
**Gram‐positive**				
*Staphylococcus aureus *(MRSA)	8	64	> 128	1
*Staphylococcus aureus *(VRSA)	8	n.d.	n.d.	16
*Streptococcus pyogenes*	32	n.d.	n.d.	n.d.

^a^ The MIC was tested by broth microdilution in triplicate. MRSA, methicillin‐resistant S. aureus; VRSA, vancomycin‐resistant S. aureus; n.d., not determined.

### 2.7 Proposed Biosynthetic Pathway for Biffamycin A (1)

**FIGURE 7:**
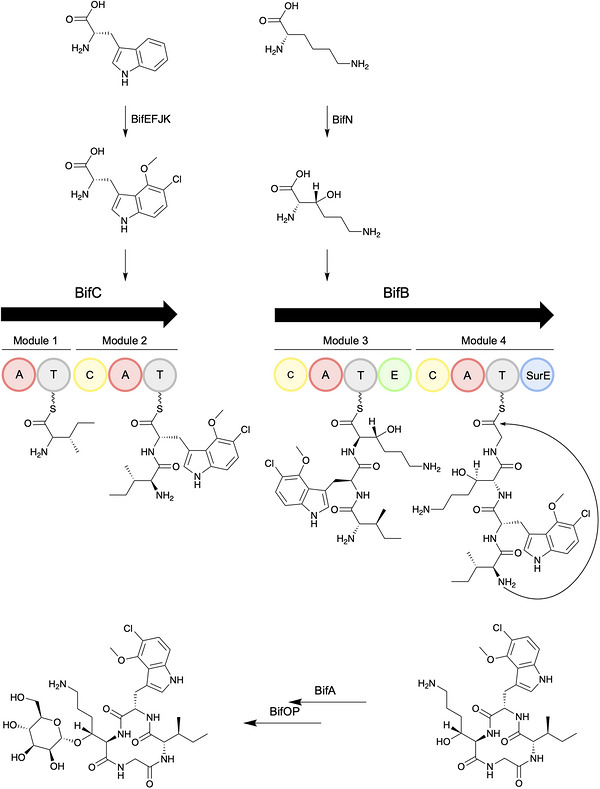
Proposed biosynthetic scheme for **1**.

Structural elucidation of **1**, combined with enzymology and bioinformatics analyses of enzymes encoded by the *bif* BGC, led us to propose a biosynthetic pathway that begins with the modification of ʟ‐Trp and ʟ‐Lys prior to utilisation as substrates for an NRPS assembly line system (Figure 7). Specifically, C4 on the ʟ‐Trp indole ring is methoxylated by the concerted action of a heme‐dependent Trp hydroxylase (BifE) and *O‐*methyltransferase (BifF). Following methoxylation, C5 of 4‐MeO‐ʟ‐Trp is chlorinated by a chlorinase/flavin reductase pair (BifKI), possibly supplied with chlorine by the putative sodium‐chlorine exchange protein, BifJ. Separately, ʟ‐Lys undergoes C3 hydroxylation catalysed by the α‐KG‐dependent hydroxylase, BifN. The cyclotetrapeptide aglycon is then assembled by the dimodular NRPSs BifC and BifB, plus BifH, which is an MbtH‐family protein that may enhance the activity of one or more of the NRPS adenylation domain(s). BifC possesses two modules organised as follows: A1‐PCP1‐C2‐A2‐PCP2. The A1 domain initiates the chain by activating and loading ʟ‐Ile onto PCP1. A2 activates and loads 5‐Cl‐4‐MeO‐ʟ‐Trp onto PCP2, which is subsequently condensed with Ile by C2. BifB possesses two modules organised as follows: C3‐A3‐PCP3‐E3‐C4‐A4‐PCP4‐SurE. A3 activates and loads 3*R*‐OH‐ʟ‐Lys onto PCP3, where it is epimerised to the D‐configuration by E3. C3 then catalyses the condensation of 3*R*‐OH‐D‐Lys with the dipeptide aminoacylthioester on PCP2. A4 activates and loads Gly onto PCP4, which is subsequently condensed with the tripeptide aminoacylthioester on PCP3 by C4 prior to macrocyclisation of the resulting tetrapeptide aminoacylthioester by the embedded SurE cyclase domain of BifB. The cyclotetrapeptide aglycon is presumably exported by the MFS1 family exporter, BifA, which shares 49% amino acid identity with MppL, an MFS1 family transporter experimentally linked to the export of the mannopeptimycin aglycon [36], where the 3*R*‐OH moiety of D‐Lys within the aglycon is mannosylated by a membrane‐bound mannosyl transferase (BifP), the substrate for which is provided by the BifO, a membrane‐bound polyprenyl‐P‐hexose synthase in a manner analogous to that proposed for mannopeptimycin, ramoplanin and teicoplanin [36, 37, 38].

### 2.8 The Antibacterial Activity of Biffamycin A (**1**)

Glycopeptide antibiotics (GPAs) are a major antibacterial class that includes cell wall biosynthesis inhibitors such as vancomycin and teicoplanin [39, 40]. GPAs are relatively large, highly crosslinked natural products and, apart from type V GPAs, possess more than one carbohydrate moiety, sometimes composed of chains up to five sugars in length [41, 42]. In this respect, **1** is remarkable because it is approximately half the size of other GPAs (i.e., 726 Da compared to 1,449 Da for vancomycin), does not contain crosslinks, and to our knowledge, is the smallest naturally occurring glycosylated cyclotetrapeptide. To evaluate whether **1** might exhibit antibacterial activity, its minimum inhibitory concentration (MIC) was determined for a small cross section of bacteria, including *Mycobacterium smegmatis* and clinical isolates of *S. aureus* resistant to methicillin (MRSA) or vancomycin (VRSA) as well as clinical isolates of *S. pyogenes, E. coli* and *P. aeruginosa*. The resulting data revealed that **1** possessed antibacterial activity against Gram‐positive organisms with an MIC of 8 µg mL^−1^ for both MRSA and VRSA, 32 µg mL^−1^ for *S. pyogenes*, and 16 µg mL^−1^ for *M. smegmatis*, whereas **1** was inactive at the concentrations used against Gram‐negative organisms tested (Table 1).

Interestingly, the biffamycin A aglycon (**6**) retained bioactivity against MRSA, albeit the MIC was 64 µg mL^−1^, eight times higher than that for **1**, while the *des‐*hydroxy biffamycin A aglycon (**3**) was inactive at 128 µg mL^−1^. At the highest concentration tested (128 µg mL^−1^), **1** did not show cytotoxicity when incubated for 48 h with human cell line HEK293 (Table 1, Figure S58). The bioactivity of **1** against only Gram‐positive bacteria and its ineffectiveness against Gram‐negative bacteria are consistent with the profile of other GPAs to which Gram‐negative bacteria possess intrinsic resistance because the molecules cannot cross the outer membrane [43]. The mechanism of action of **1** remains to be determined; however, given that it is not cytotoxic to HEK293 cells, it is tempting to speculate **1** may target the Gram‐positive cell envelope, like other GPAs [44]. Excitingly, the ability of **1** to inhibit the growth of MRSA equally well as VRSA indicates that **1** either has a mechanism of action different from vancomycin or that it circumvents VanA‐mediated resistance.

## 3 Conclusion

Antibiotic resistance poses a severe threat to global health. While most antibiotics are derived from microbial natural products, many, if not most, biosynthetic pathways are not expressed or productive in the laboratory. To overcome this challenge, we used promoter engineering to de‐regulate the expression of a silent biosynthetic gene cluster, which enabled isolation and characterisation of its product—a novel glycopeptide antibiotic (GPA) we named biffamycin A. Biffamycin A contains two unprecedented chemical moieties, 3‐hydroxy(α‐D‐mannoysl)‐D‐Lys and 5‐chloro‐4‐methoxy ʟ‐Trp, whose biosynthesis we characterised through in vitro and in vivo reconstitution experiments. SurE/PBP‐TE cyclases are emerging as biocatalysts to improve yield and diversity during chemical synthesis of cyclopeptide therapeutics [45, 46, 47]. A particular challenge is the cyclisation of tetrapeptides where current methods are inadequate [48, 49]. BifB^SurE^ is the first member of the SurE/PBP‐TE family whose native substrate is a tetrapeptide, which together with a recently characterised hexapeptide cyclase with tetrapeptide cyclase activity [50], provides a foundation to generate diverse libraries of cyclotetrapeptides for drug discovery. Moreover, biffamycin A stands out as the smallest known member of its class, lacking the complex structural features typically associated with GPAs. Despite its simplicity, biffamycin A demonstrates potent activity against drug‐resistant bacteria, including MRSA and VRSA, as well as the model organism, *M. smegmatis*. This discovery presents a unique opportunity to leverage the scaffold of biffamycin A for developing novel therapeutics against *M. tuberculosis*, a pathogen of critical priority for the World Health Organization [51].

The authors have cited additional references within the Supporting Information [18, 27, 52, 53, 54, 55, 56, 57, 58, 59, 60, 61, 62, 63, 64, 65, 66, 67, 68, 69, 70, 71, 72, 73, 74, 75], which contains materials and methods, and additional figures and tables. Cartesian coordinates for all DFT‐calculated peptide conformations (**1**, **6**, and **7**) and calculated chemical shift tables are available in compressed, downloadable electronic format.

## Conflicts of Interest

The authors declare no conflicts of interest.

The authors declare no conflicts of interest.

## Supporting information




**Supporting File 1**: anie72473‐sup‐0001‐SuppMat.docx.

**Supporting File 2**: anie72473‐sup‐0002‐TableS1.xlsx.

**Supporting File 3**: anie72473‐sup‐0003‐Data.zip.

## Data Availability

The data that support the findings of this study are available in the supplementary material of this article.
