# Supplementary figures and images for: Discovery and Biosynthesis of the Novel Glycotetrapeptide Antibiotic Biffamycin A
Source: Angew Chem Int Ed Engl. 2026 May 6;65(26):e11349. doi: 10.1002/anie.202511349 (PMC13285481; doi:10.1002/anie.202511349)

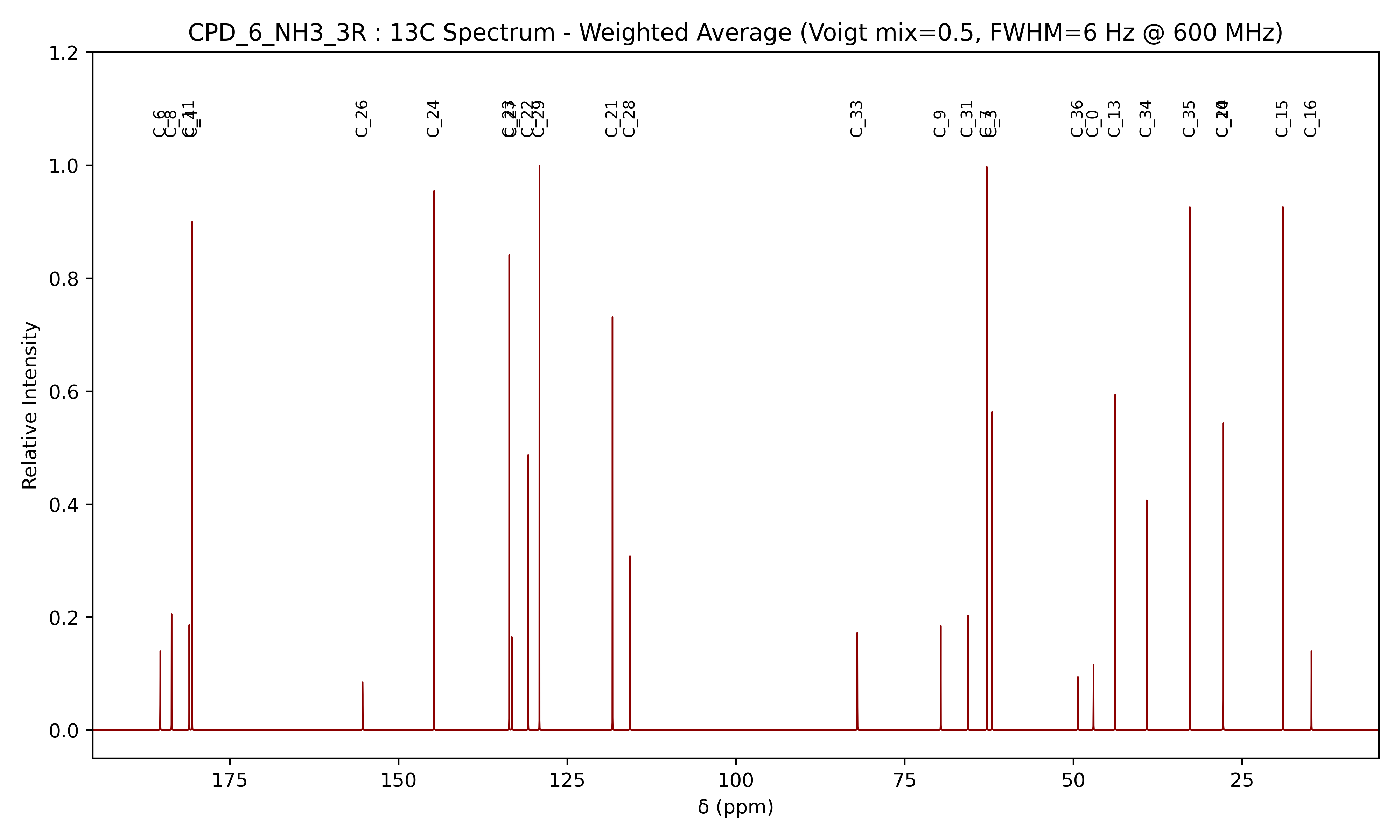

Supplement: Supplementary file 3 — Supporting File 3: anie72473‐sup‐0003‐Data.zip. [file ANIE-65-e11349-s002.zip › r2SCAN3c_ensembles/6_3R/CPD_6_NH3_3R_NMR_spectrum_C_weighted_average.png]

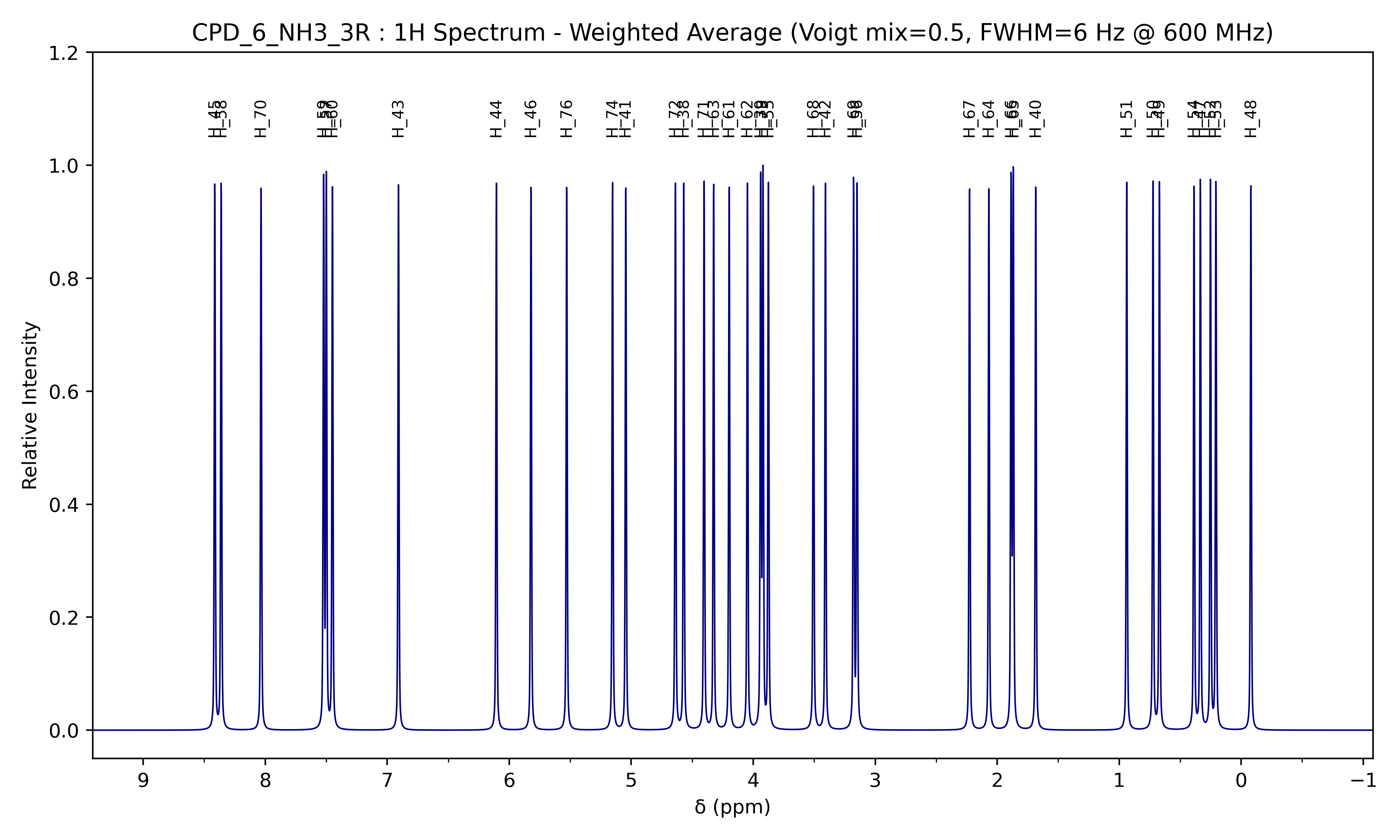

Supplement: Supplementary file 3 — Supporting File 3: anie72473‐sup‐0003‐Data.zip. [file ANIE-65-e11349-s002.zip › r2SCAN3c_ensembles/6_3R/CPD_6_NH3_3R_NMR_spectrum_H_weighted_average.png]

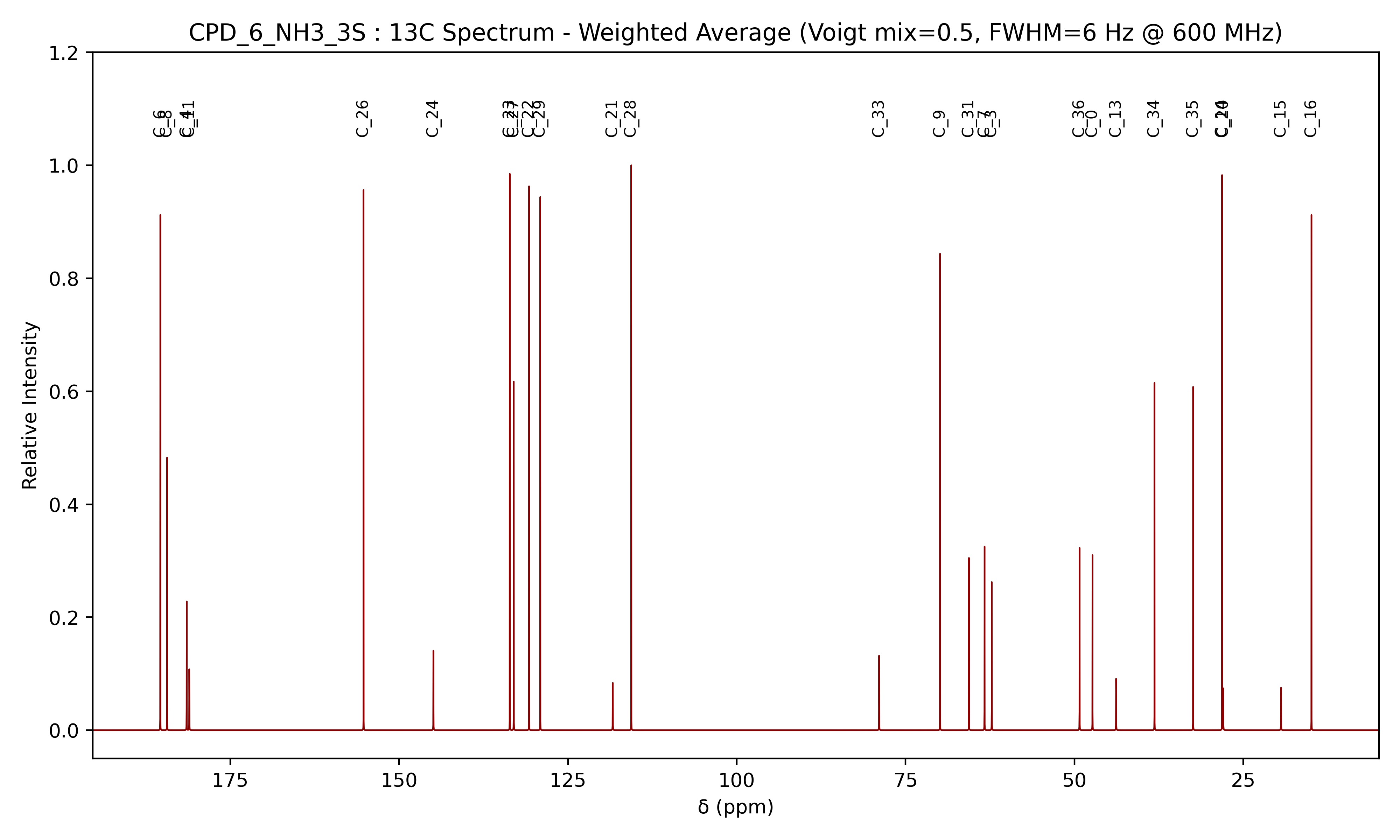

Supplement: Supplementary file 3 — Supporting File 3: anie72473‐sup‐0003‐Data.zip. [file ANIE-65-e11349-s002.zip › r2SCAN3c_ensembles/6_3S/CPD_6_NH3_3S_NMR_spectrum_C_weighted_average.png]

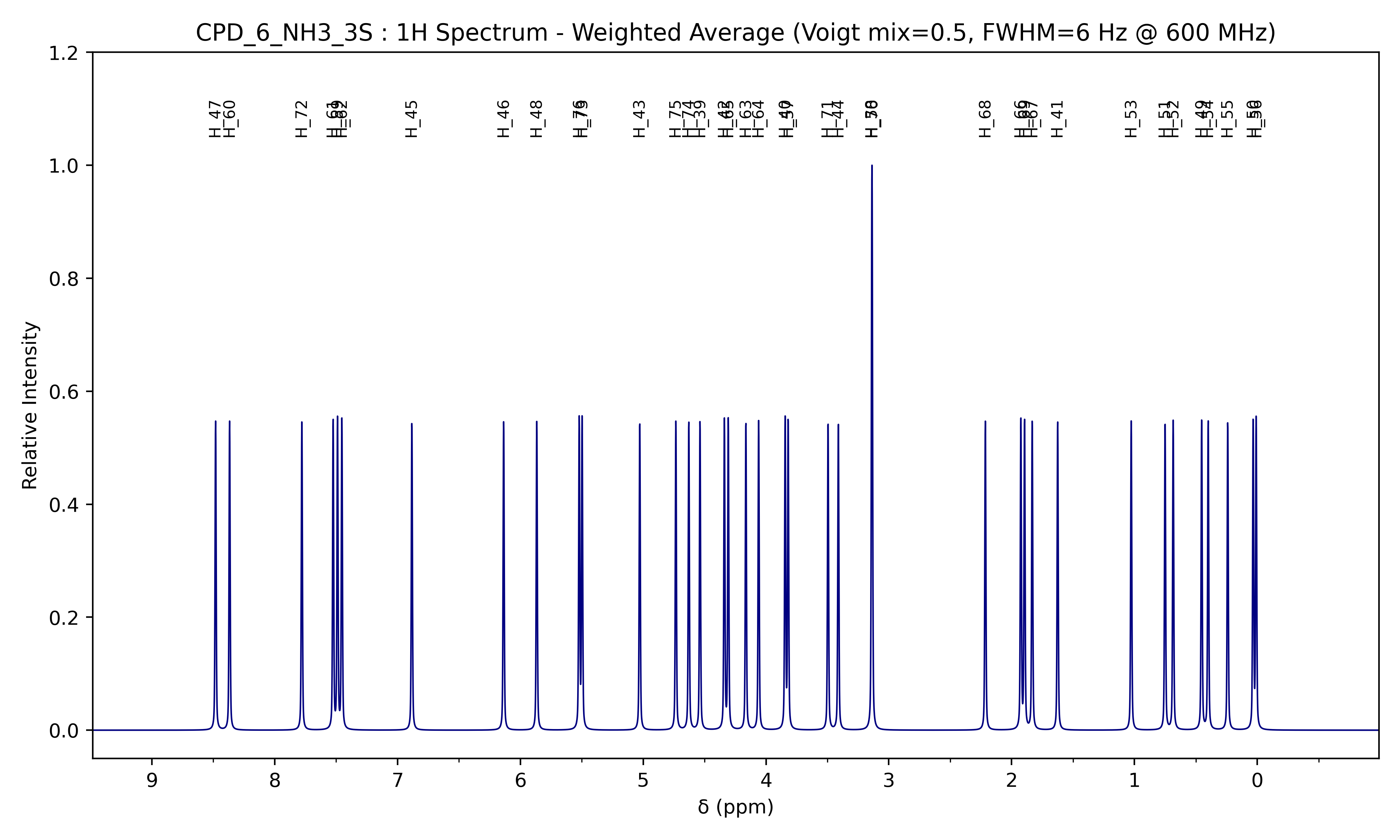

Supplement: Supplementary file 3 — Supporting File 3: anie72473‐sup‐0003‐Data.zip. [file ANIE-65-e11349-s002.zip › r2SCAN3c_ensembles/6_3S/CPD_6_NH3_3S_NMR_spectrum_H_weighted_average.png]

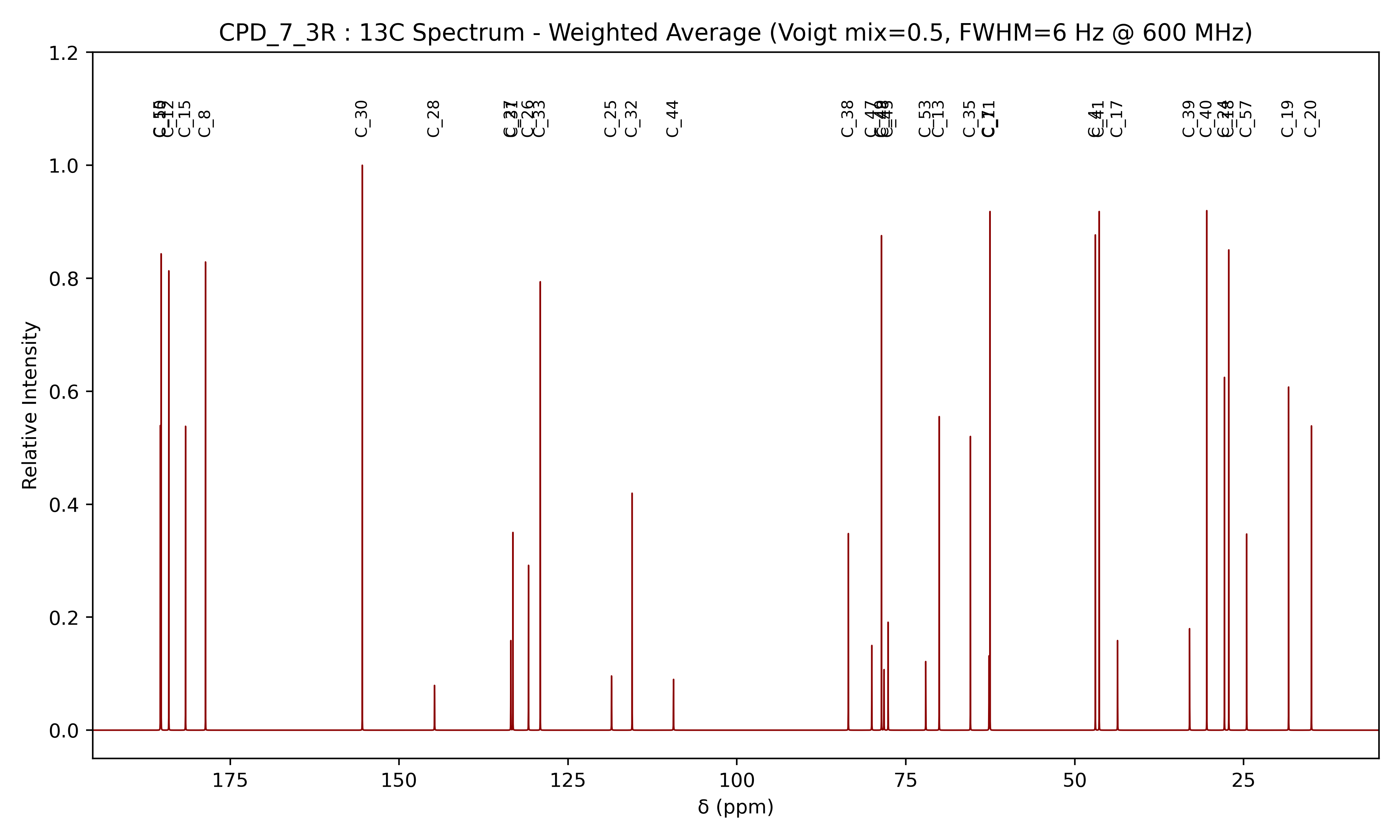

Supplement: Supplementary file 3 — Supporting File 3: anie72473‐sup‐0003‐Data.zip. [file ANIE-65-e11349-s002.zip › r2SCAN3c_ensembles/7_3R/CPD_7_3R_NMR_spectrum_C_weighted_average.png]

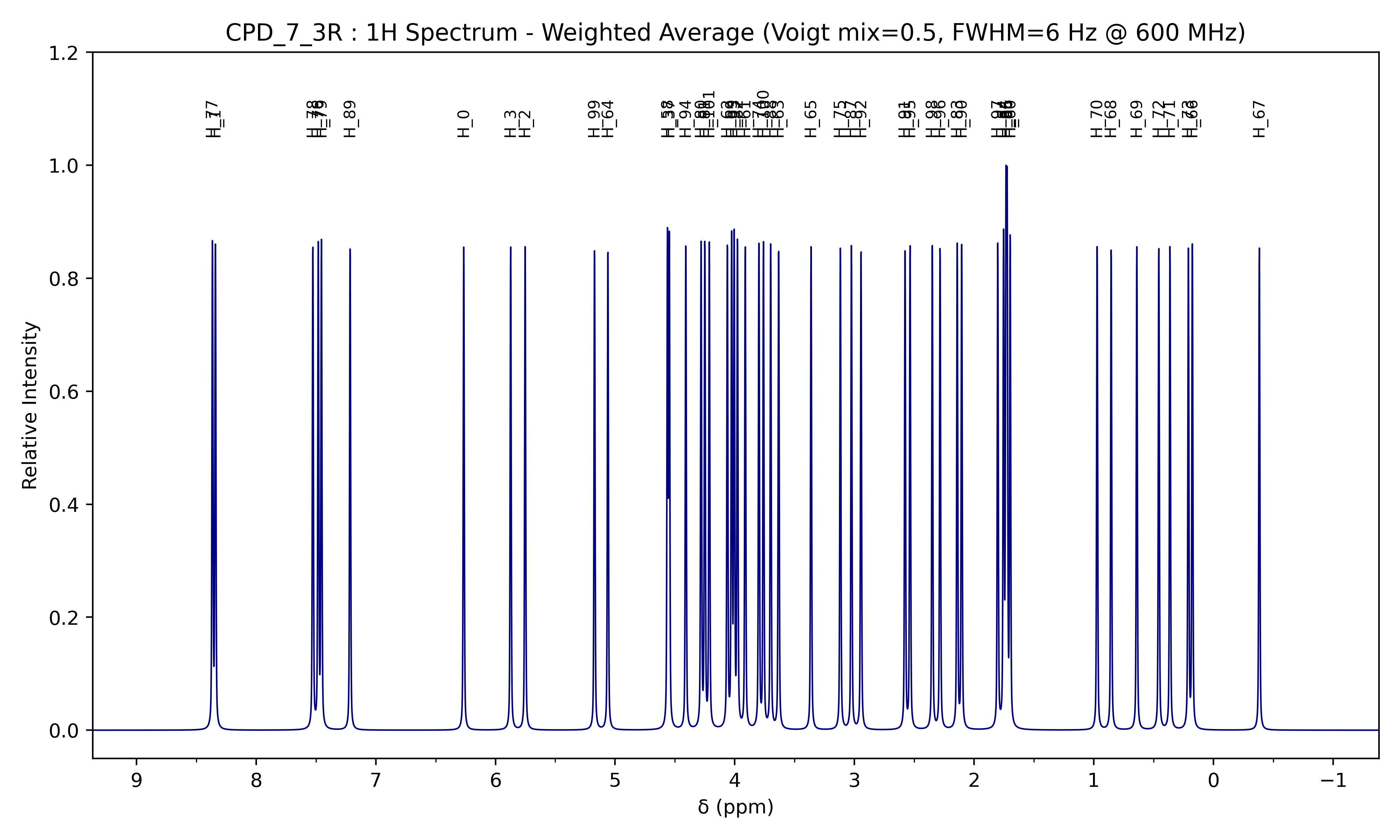

Supplement: Supplementary file 3 — Supporting File 3: anie72473‐sup‐0003‐Data.zip. [file ANIE-65-e11349-s002.zip › r2SCAN3c_ensembles/7_3R/CPD_7_3R_NMR_spectrum_H_weighted_average.png]

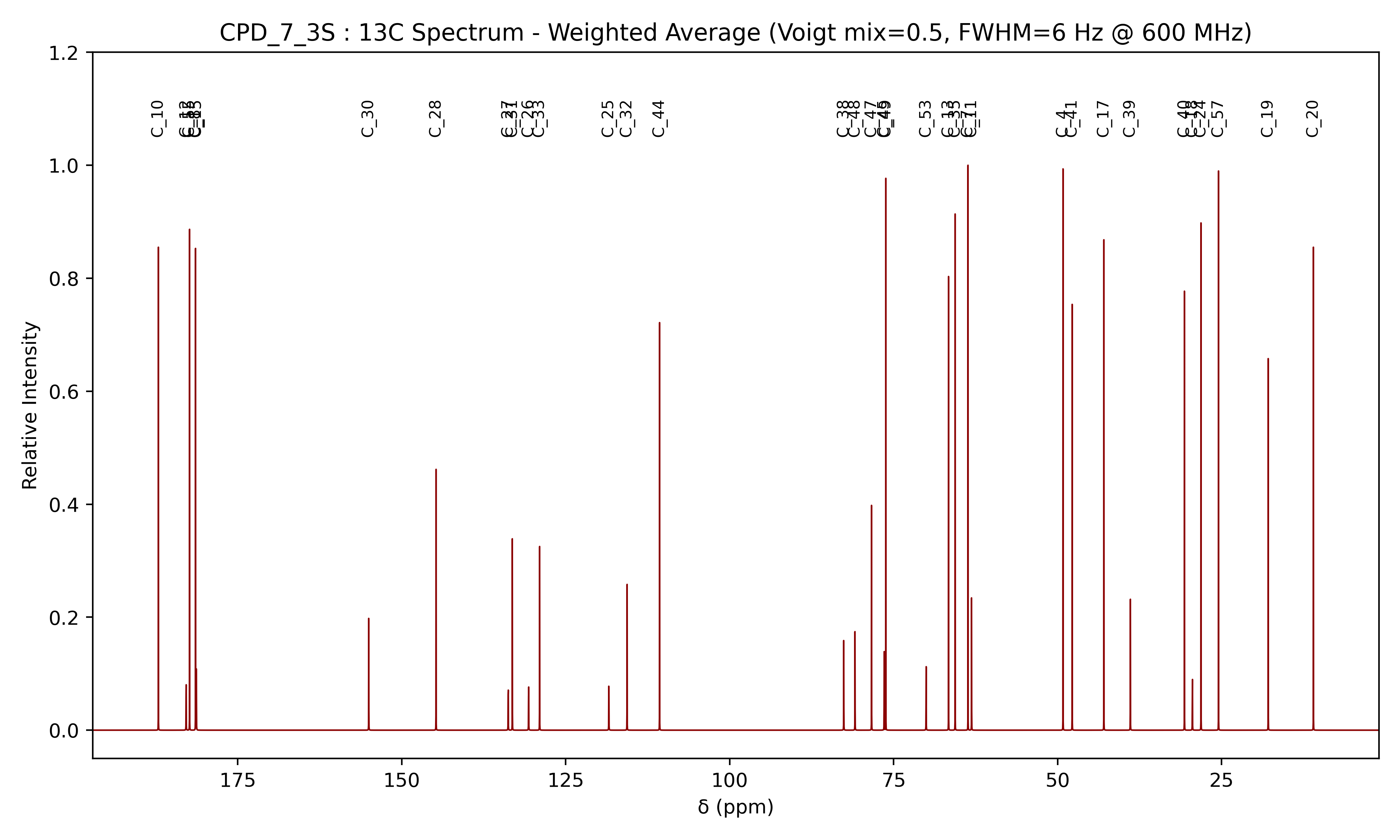

Supplement: Supplementary file 3 — Supporting File 3: anie72473‐sup‐0003‐Data.zip. [file ANIE-65-e11349-s002.zip › r2SCAN3c_ensembles/7_3S/CPD_7_3S_NMR_spectrum_C_weighted_average.png]

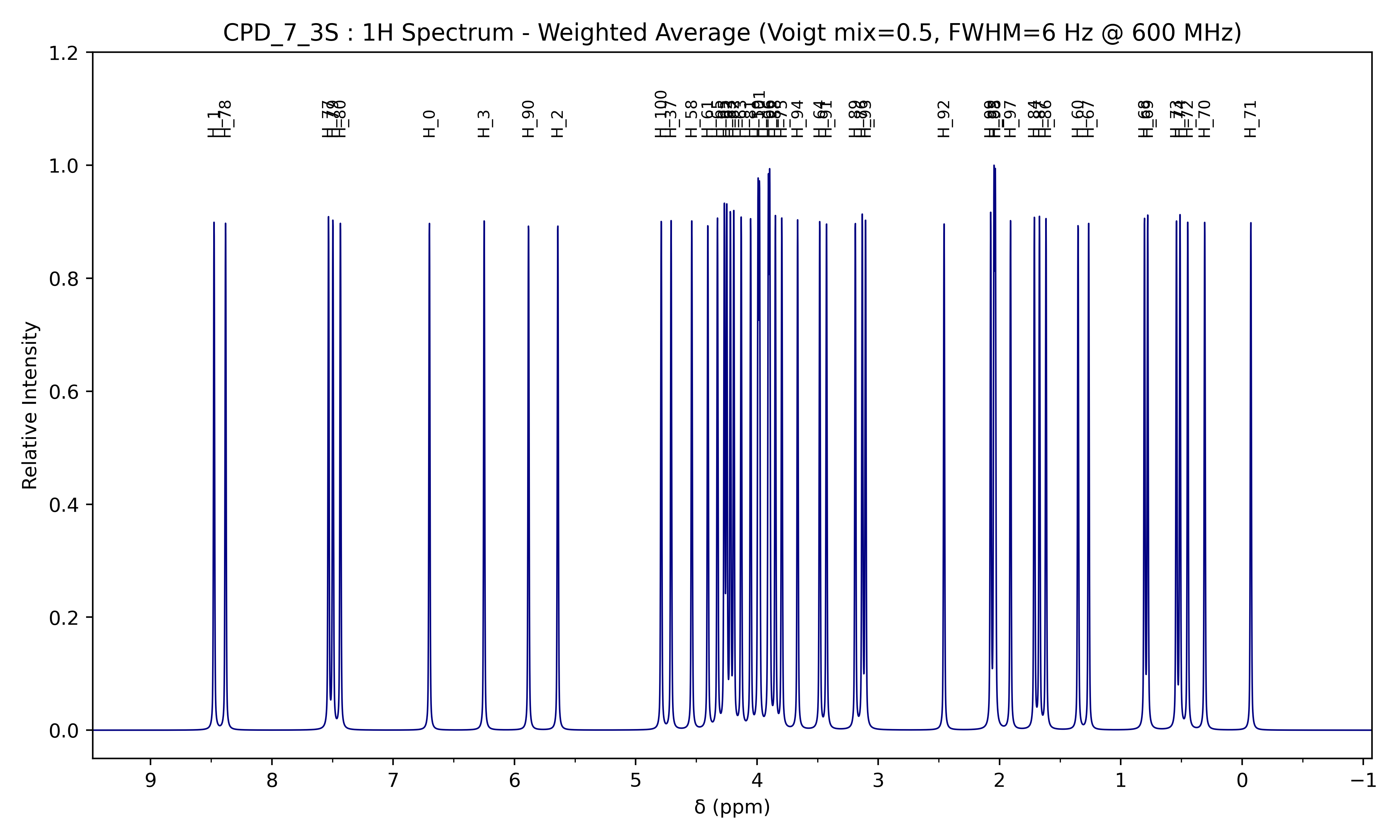

Supplement: Supplementary file 3 — Supporting File 3: anie72473‐sup‐0003‐Data.zip. [file ANIE-65-e11349-s002.zip › r2SCAN3c_ensembles/7_3S/CPD_7_3S_NMR_spectrum_H_weighted_average.png]
